# Ultrastructural Characterization of Human Oligodendrocytes and Their Progenitor Cells by Pre-embedding Immunogold

**DOI:** 10.3389/fnana.2021.696376

**Published:** 2021-06-23

**Authors:** María J. Ulloa-Navas, Pedro Pérez-Borredá, Raquel Morales-Gallel, Ana Saurí-Tamarit, Patricia García-Tárraga, Antonio J. Gutiérrez-Martín, Vicente Herranz-Pérez, Josée M. García-Verdugo

**Affiliations:** ^1^Laboratory of Compared Neurobiology, University of Valencia-CIBERNED, Paterna, Spain; ^2^Neurosurgery Department, Consorcio Hospital General Universitario de Valencia, Valencia, Spain; ^3^Neurosurgery Department, Hospital Universitari i Politècnic La Fe, Valencia, Spain; ^4^Predepartmental Unit of Medicine, Faculty of Health Sciences, Universitat Jaume I, Castelló de la Plana, Spain

**Keywords:** oligodendrocytes, OPCs, human oligodendrocytes, transmission electron microscopy, immunogold, BCAS1

## Abstract

Oligodendrocytes are the myelinating cells of the central nervous system. They provide trophic, metabolic, and structural support to neurons. In several pathologies such as multiple sclerosis (MS), these cells are severely affected and fail to remyelinate, thereby leading to neuronal death. The gold standard for studying remyelination is the g-ratio, which is measured by means of transmission electron microscopy (TEM). Therefore, studying the fine structure of the oligodendrocyte population in the human brain at different stages through TEM is a key feature in this field of study. Here we study the ultrastructure of oligodendrocytes, its progenitors, and myelin in 10 samples of human white matter using nine different markers of the oligodendrocyte lineage (NG2, PDGFRα, A2B5, Sox10, Olig2, BCAS1, APC-(CC1), MAG, and MBP). Our findings show that human oligodendrocytes constitute a very heterogeneous population within the human white matter and that its stages of differentiation present characteristic features that can be used to identify them by TEM. This study sheds light on how these cells interact with other cells within the human brain and clarify their fine characteristics from other glial cell types.

## Introduction

Oligodendrocytes are glial cells whose main function is myelination. Myelination leads to the optimization of the saltatory conduction along neuronal axons (McLaurin and Yong, 1995). Moreover, oligodendrocytes perform other supportive activities such as glucose metabolism (Amaral et al., 2016; Saab et al., 2016), calcium influx/efflux towards the axon to impede degeneration (Witte et al., 2019), and pH and ion balance regulation (Inouye and Kirschner, 1988). Given that oligodendroglial or myelin degeneration can produce quite severe pathologies such as in genetic conditions in children (Greenfield, 1933; Renier et al., 1981; Matalon et al., 1988; van der Knaap et al., 1995; Tappino et al., 2010) or neurodegenerative conditions with a very high incidence in adults such as multiple sclerosis (MS; Howard et al., 2016) and that the causes that may trigger oligodendroglial damage can be quite diverse e.g., genetic (Greenfield, 1933; Renier et al., 1981; Matalon et al., 1988; Tappino et al., 2010; van der Knaap et al., 1995), autoimmune (Howard et al., 2016), infectious (Stohlman and Hinton, 2001), and vascular (Volpe, 2001; Chavali et al., 2020)—studying the biology underlying this cell population and its progenitors is a relevant aspect in central nervous system research.

After numerous studies in animal models, evidence suggests that in the postnatal brain oligodendrocytes arise from NG2/PDGFRa/Olig2-positive progenitors in different regions of the brain and spinal cord (Rivers et al., 2008; Zhu et al., 2008; Kang et al., 2010; Sánchez-González et al., 2020). The processes of oligodendrocyte replacement, maturation, and myelin regeneration play a critical role in the adult brain, where remodeling and plasticity are a constant phenomenon. Correspondingly, when there is an insult in the brain, especially in the white matter such as in MS, NG2^+^-cells are recruited towards the lesions, and thus, studies demonstrate their presence in MS plaques (Wilson et al., 2006).

In rodents, the stages of differentiation of the oligodendroglial lineage have been largely studied and have shed light on the expression of different markers of oligodendrocyte maturation (Barateiro and Fernandes, 2014; Marques et al., 2016; Domingues et al., 2018; Schoor et al., 2019). These studies have described that there are at least three oligodendrocyte stages according to the morphology and protein markers expressed by each population. The first population corresponds to oligodendrocyte precursor cells (OPCs), which express markers such as: NG2, PDGFRα, A2B5, CD9, CNP, GPR17, NKX 2.2, Olig1, Olig2, Sox9, and Sox10. The second population is pre-oligodendrocytes or premyelinating oligodendrocytes (pre-OLs) which can be labeled for CD9, O4, BCAS1, CNP; NKX 2.2, Sox17, Sox10, and Olig2. The last population is myelinating oligodendrocytes (m-OLs) which express: O1, CD9, CNP, MAG, MBP, MOG, PLP1, Sox17, APC1-(CC1), and Olig2 (Goldman and Kuypers, 2015). Furthermore, single-cell transcriptional analysis has described 12 different populations of oligodendrocytes and their progenitors along with the juvenile and adult postnatal brain, thereby describing the great heterogeneity among these cells (Marques et al., 2016; van Bruggen et al., 2017). However, the fine morphology of oligodendrocytes is still elusive.

In contrast to mice, the number of human oligodendrogenesis studies is not as high. This can be explained because of the scarce availability of human brain tissue and poor fixation conditions. This is more evident in ultrastructural studies, which require an outstanding fixation and tissue preservation. Considering that the gold standard for understanding remyelination is the g-ratio measured by transmission electron microscopy (TEM), analyzing human tissue using TEM is fundamental.

**Figure 1:**
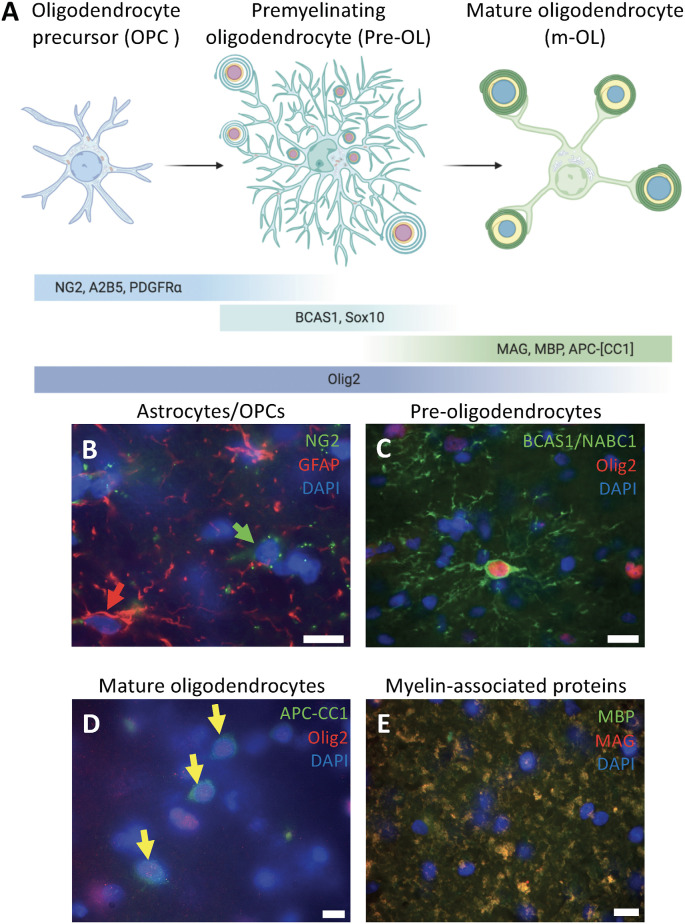
Differentiation stages of oligodendrocytes in human white matter. **(A)** Schematic diagram of the oligodendrocyte differentiation process representing the morphology, organelle distribution, and markers to identify each of the three stages: oligodendrocyte precursor cells (OPCs), premyelinating oligodendrocytes (pre-OLs), and mature oligodendrocytes. **(B)** Human white matter labeled for the astrocytic marker GFAP (red arrow) and the OPC marker NG2 (green arrow) showing that these are two different populations within the white matter of a 39-year-old male (PB4). **(C)** Human white matter labeled for pre-myelinating oligodendrocyte markers BCAS1/NABC1 and Olig2 co-expressed in highly ramified cells from a 5-year-old female (PB19). **(D)** Mature oligodendrocytes labeled for Olig2 and APC-(CC1; yellow arrows) from a white matter sample of a 5-year-old female (PB19). **(E)** Myelin wrapped axons co-expressing MAG and MBP from a white matter sample of a 39-year-old male (PB4). Scale bars: 50 μm.

Here we present a detailed study of the fine structure of oligodendrocytes in different stages in human white matter. This analysis includes samples from patients at different ages with exceptional preservation of the tissue combined with pre-embedding immunogold for nine different markers. Our results suggest that oligodendrocytes present three marked ultrastructural stages (Figure 1A): OPCs labeled for NG2, A2B5 and PDGFRα; pre-OLs labeled for BCAS1/NABC1, Sox10 and Olig2; and m-OLs labeled with APC-(CC1) and Olig2. Furthermore, we studied the presence of myelin-associated proteins and myelin ultrastructure labeling for MAG and MBP combined with standard TEM.

## Materials and Methods

All reagents were purchased from Sigma Aldrich unless otherwise specified.

### Human Sample Collection

Samples were obtained from surgical resections from focal cortical dysplasia patients operated at Hospital Universitarii Politècnic La Fe at Valencia, Spain. Samples included in this study (*n* = 10) comprehend the non-affected white matter of female and male patients ages 2–44-year-old (Table 1). Sample collection was approved by the Research Ethics committee from the Hospital Universitari i Politècnic La Fe. Patients or their representatives signed an informed consent prior to donating the samples for the study.

**Table 1:** Human samples and brain areas included in this study.

**Sample code**	**Age (years)**	**Sex**	**Diagnosis**	**Area**
PB2	9	Male	Type IA FCD	Left temporal lobe
PB3	6	Male	Type IA FCD	Left temporal lobe
PB4	39	Male	Type IIB FCD	Left frontal lobe
PB8	27	Male	Type IIA FCD	Right anterior insular cortex
PB9	2	Female	Type IIB FCD	Right temporal lobe
PB11	13	Female	Type IC FCD	Left frontal lobe
PB12	44	Male	Type IIB FCD	Right frontal lobe
PB16	32	Male	Type IB FCD	Right frontal lobe
PB19	5	Female	No lesion	Right frontal lobe
PB20	2	Female	Type IIA FCD	Right frontal lobe

*Note: FCD, focal cortical displasia*.

### Sample Processing

After surgical resection samples were immediately fixed with 4% paraformaldehyde in 0.1 M phosphate buffer (pH 7.4, PB) for 7 days. Samples were then washed with 0.1 M PB and kept in 0.1 M PB-0.05% sodium azide at 4°C until subsequent use. Brain tissue was sectioned at 50 μm using a Leica VT1000S vibratome (Leica Biosystems, Wetzlar, Germany). We performed the same procedures in a P21 4% PFA perfused mouse as a control for the following experiments, except for A2B5 and PDGFRa immunogold labeling, since the antibodies worked for human tissue only (Table 2).

**Table 2:** Antibody and concentrations used.

**Antigen**	**Host species**	**Antibody**	**IF concentration**	**IG concentration**	**Cell type labeling**
NG2	Rabbit	ab5320 (Abcam)	1:200	1:100	OPCS
PDGFRα	Mouse	556001 (BD Biosciences)	1:50	1:50	OPCs
A2B5	Mouse	MAB312 (Millipore)	1:100	1:50	OPCS
Olig2	Rabbit	AB9610 (Millipore)	1:300	1:150	OPCs, pre-OLs, m-OLs
BCAS1	Mouse	sc-136342 (Santa Cruz)	1:200	1:150	pre-OLs
Sox10	Rabbit	ab155279 (Abcam)	1:300	1:150	pre-OLs
APC (CC1)	Mouse	ab16794 Abcam	1:100	1:50	m-OLs
MAG	Mouse	Mab15667 (Millipore)	1:100	1:100	myelin
MBP	Rat	ab7349 (Abcam)	1:100	1:100	myelin
GFAP	Mouse	Millipore	1:200	1:100	Astrocytes

### Immunofluorescence

Sections were washed with 0.1 M PB, incubated in 1:200 Immunosaver (Electron Microscopy Sciences, Hatfield, PA) in water at 60°C for 30 min. Peroxidase blocking was performed using a solution containing 10% methanol and 10% H_2_O_2_ in 0.1 M PB. For permeabilization, the samples were washed three times for 5 min in PTA solution: 0.1% Triton X-100, 1 mg/ml bovine serum albumin (BSA) in 0.1 M phosphate buffer saline (PBS). The samples were then incubated in blocking solution (5% normal goat serum in PTA) for 1 h at room temperature. Subsequently, the samples were incubated overnight in primary antibodies diluted in blocking solution [1:300 rabbit anti-Olig2, Merck-Millipore Burlington, MA; 1:200 rabbit anti-NG2, Abcam, Cambridge, United Kingdom; 1:200 mouse anti-GFAP, Merck-Millipore Burlington, MA; 1:200 mouse anti-NABC1, Santa Cruz Biotechnology, Dallas, TX; 1:100 mouse anti-MAG, Merck-Millipore Burlington, MA; 1:100 rat anti MBP, Abcam, Cambridge, United Kingdom, 1:100 mouse anti-APC (CC1), Abcam, Cambridge, United Kingdom; see Table 3]. The following day, samples were thoroughly washed in PTA and incubated in a secondary antibody solution (1:500 AlexaFluor 488 goat anti-mouse, Invitrogen; or 1:500 AlexaFluor 555 goat anti-rabbit, Invitrogen). Samples were then washed in 0.1 M PB and incubated in 1:1,000 DAPI in water for 5 min. Finally, the samples were mounted with FluorSave [Merck Millipore (Calbiochem), Burlington, MA].

**Table 3:** Antibody tested per sample.

**Antigen**	**NG2**	**PDGFRα**	**A2B5**	**Olig2**	**BCAS1**	**Sox10**	**APC-(CC1)**	**MAG**	**MPB**	**GFAP**
**PB2**										
**PB2**	
**PB4**										
**PB8**										
**PB9**	
**PB11**										
**PB12**										
**PB16**										
**PB19**			
**PB20**										
**Mouse**										

*Shaded areas correspond to antibodies tested in the samples indicated*.

### Immunogold Labeling

Tissue sections were cryoprotected in a solution containing 25% saccharose in 0.1 M PB-0.05% azide for 30 min. Immediately after cryoprotection, sections were permeabilized by repeated freeze–thaw cycles by immersion in −60°C 2-methylbutane and rapidly transfer to a room temperature saccharose solution. These steps were repeated three or four times. Subsequently, tissue sections were left in 0.1 M PB and then incubated in primary antibody blocking solution [0.3% BSAc (Aurion, Wageningen, The Netherlands), 0.05% sodium azide in 0.1 M PB] for 1 h. Next, the samples were incubated in primary antibody [1:150 rabbit anti-Olig2, Merck-Millipore Burlington, MA; 1:100 mouse anti-GFAP, Merck-Millipore Burlington, MA; 1:100 mouse anti-MAG, Merck-Millipore Burlington, MA; 1:100 rat anti MBP, Abcam, Cambridge, United Kingdom, 1:50 mouse anti-APC (CC1), Abcam, Cambridge, United Kingdom; 1:50 mouse anti-A2B5 Merck-Millipore Burlington, MA, 1:150 rabbit anti-Sox10 Abcam, Cambridge, United Kingdom; see Table 3] in primary antibody blocking solution for 72 h at 4°C. The samples were rinsed in 0.1 M PB and then incubated in secondary antibody blocking solution consisting of 0.5% BSAc (Aurion), 0.025% CWFS gelatin (Aurion), 0.05% sodium azide in 0.1 M PB for 1 h, followed by incubation in secondary antibody [1:50 goat anti-mouse IgG gold ultrasmall (Aurion); 1:50 goat anti-rabbit IgG gold ultrasmall (Aurion); 1:50 goat-anti-rat IgG gold ultrasmall (Aurion)] diluted in the same secondary antibody blocking solution overnight at 4°C. To enhance gold labeling, we performed silver enhancement (R-GENT SE-LM, Aurion) for 15–25 min in the dark, followed by gentle washing in 2% sodium acetate and incubation in gold toning solution (0.05% gold chloride in water) for 10 min. The samples were then washed twice with 0.3% sodium thiosulfate in water. Finally, we post-fixed with 2% glutaraldehyde (Electron Microscopy Sciences) in 0.1 M PB for 30 min. Samples were rinsed and kept in 0.1 M PB containing 0.05% sodium azide at 4°C until processing them for resin embedding.

For PDGFRa, NABC1, and NG2, we performed Tyramide Signal Amplification. Sections were washed in 0.05% Triton X-100 in PBS (PBS-Tx) at room temperature for 15 min. To quench endogenous peroxidases, we incubated the sections in peroxidase blocking solution (1% H_2_O_2_ in PBS-Tx) for 45 min in the dark at room temperature. Samples were washed and incubated in blocking solution [0.87% NaCl, 5% normal goat serum (Gibco), 0.05% sodium azide in 0.1 M Tris-HCl at pH 7.5] for 1 h. Then, the sections were incubated in primary antibody overnight (1:50 mouse anti-PDGFRa, BD Biosciences, Franklin Lakes, NJ; 1:100 rabbit anti-NG2 Abcam, Cambridge, United Kingdom, 1:150 mouse anti-NABC1 Santa Cruz Biotechnology, Dallas, TX; see Table 3) under mild agitation at 4°C. Samples were subsequently washed in PBS-Tx and incubated in secondary antibody solution for 2 h at room temperature. After washing with PBS-Tx, an incubation in Streptavidin-HRP (Vector Labs, San Francisco, CA) solution was performed for 30 min. Then, we incubated the samples in 1:50 biotin-tyramide solution (Akoya Biosciences, Marlborough, MA) for 5 min at room temperature. Subsequently, the sections were blocked in 0.3% BSAc (Aurion, The Netherlands) in 0.1 M PB-0.05% sodium azide for 1 h at room temperature in the dark. Then we incubated the samples in immunogold primary antibody (1:300 mouse anti-Biotin antibody; Jackson Immunoresearch) diluted in blocking solution overnight at 4°C with gentle shaking. The following day, samples were washed and incubated in a blocking solution consisting of 0.5% BSAc and 0.1% fish skin gelatin (Aurion) in 0.1 M PB-0.05% sodium azide. Next, we incubated the samples in 1:50 goat anti-mouse gold-conjugated antibody (Ultra Small; Aurion) in blocking solution for 2 h, in the dark at and under mild agitation. Finally, we proceeded with the silver enhancement process as in the standard immunogold labeling as described above.

### Tissue Processing for TEM Analysis

For TEM analysis, sections were embedded in epoxy resin. First, samples were post-fixed with 1% osmium tetroxide (Electron Microscopy Sciences), 7% glucose in 0.1 M PB for 30 min at room temperature, washed in deionized water, and partially dehydrated in 70% ethanol. Afterward, the samples were contrasted in 2% uranyl acetate (Electron Microscopy Sciences) in 70% ethanol for 2 h at 4°C. The samples were further dehydrated and embedded in Durcupan ACM epoxy resin at room temperature overnight, and then at 70°C for 72 h. Once the resin was polymerized, immunolabeled sections were selected and cut into semithin (1.5 μm) and then into ultrathin sections (60–80 nm) using an Ultracut UC7 ultramicrotome (Leica). Ultrathin sections were placed on formvar-coated single-slot copper grids (Electron Microscopy Sciences) stained with lead citrate and examined at 80 kV on a FEI Tecnai G^2^ Spirit (FEI Company, Hillsboro, OR) transmission electron microscope equipped with a Morada CCD digital camera (Olympus, Tokyo, Japan).

### Criteria Used for Immunogold Analysis

Our general criteria to differentiate label and background is 5:1 ratio of silver-enhanced gold nanoparticles. And depending on the labeled structure and marker (plasma membrane or organelle) we consider a minimum number of silver-enhanced nanoparticles within an ultrathin section as follows:

For NG2: seven silver-enhanced gold nanoparticles/μm^2^.

PDGFRα: seven silver-enhanced gold nanoparticles/cluster in at least three clusters per cell.

A2B5: seven silver-enhanced gold nanoparticles/μm^2^ when there is a proximal ER.

BCAS1: 10 silver-enhanced gold nanoparticles within the extent of the plasma membrane.

Olig2: at least 15 silver-enhanced gold nanoparticles within the nucleus.

Sox10: at least 15 silver-enhanced gold nanoparticles within the nucleus.

APC: at least 15 silver-enhanced gold nanoparticles within the cell.

MAG/MBP: at least 15 silver-enhanced gold nanoparticles in the myelin proximity.

GFAP: 15 silver-enhanced gold nanoparticles/μm^2^.

## Results

### Human Oligodendrocytes Go Through Three Sequential Morphological Stages of Differentiation

Previous studies indicate that human oligodendrocytes acquire three sequential phenotypes through their maturation (Goldman and Kuypers, 2015). The identification of these maturational stages is typically based on the expression of different molecular markers. In order to establish a connection between the molecular and ultrastructural changes which characteristically follow each maturational stage, we first performed immunofluorescence detection for a group of markers described for oligodendrocytes, their progenitors, and myelin proteins. This analysis was performed in human intraoperatively obtained brain tissue containing white matter from donors of different ages. Our results suggest that oligodendrocytes in the human white matter display three distinctive morphologies and can be labeled with distinctive molecular markers (Figure 1A). The first stage is characterized by star-shaped cells, negative for astrocyte marker GFAP, in which NG2-labeling is distributed as cytoplasmic/plasma membrane dots in cells evenly distributed through the white matter (Figure 1B). NG2 expression defines a population which has been widely described as OPCs with a broad distribution through the white matter. The second stage of differentiation was identified with the oligodendrocyte lineage markers BCAS1/NABC1 and Olig2, corresponding to pre-OLs. Pre-OLs expand their processes and become highly ramified cells with a small soma (Figure 1C). The subcellular localization of Olig2 is limited to the cell nucleus, while in the case of BCAS1/NABC1 it is cytoplasmic. The third and final stage of differentiation comprises m-OLs, which are small cells with scant cytoplasm distributed throughout the white matter and label profusely for the nuclear transcription factor Olig2 and the RNA binding protein quaking7, which binds to APC-(CC1) antibody. This protein appears to be in a cytoplasmic/nuclear localization when detected by immunofluorescence (Figure 1D). Finally, we have observed myelin-associated proteins such as MAG and MBP broadly scattered through the white matter (Figure 1E). Our results confirm that at least three maturational stages can be defined in the oligodendroglial lineage based on molecular marker expression and gross morphology.

### OPCs in the Human White Matter Display an Astrocytic-like Morphology and Ultrastructure

**Figure 2:**
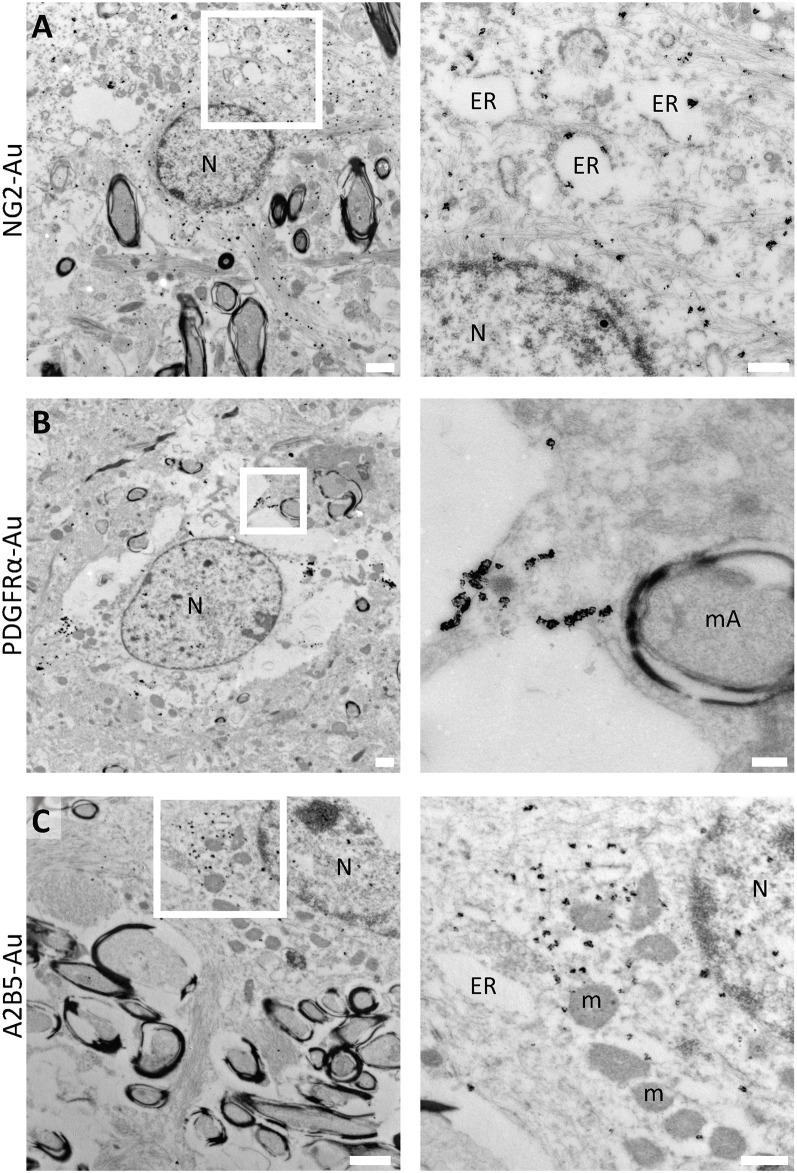
Immunoelectron microscopy for human OPC molecular markers. **(A)** NG2 label in large electron-lucent cells in the white matter. Inset shows NG2 subcellular distribution in cytoplasmic intermediate filaments and in short dilated endoplasmic reticulum cisternae in a sample of a 2-year-old male sample (PB20). **(B)** PDGFRα is expressed in discrete clusters in the plasma membrane of OPCs in the white matter of a 6-year-old male (PB3). **(C)** OPCs in the white matter labeled with A2B5. Immunogold shows the subcellular localization of this marker in the cytoplasmic portion of the cells and is also associated with the endoplasmic reticulum in the white matter of a 5-year-old female (PB19). N, Nucleus; ER, endoplasmic reticulum; mA, myelinated axon; m, mitochondria. Scale bars: panoramic micrographs, 1 μm; insets: 250 nm.

To better characterize the ultrastructure of human OPCs, we have analyzed cells labeled for NG2, PDGFRα, A2B5, and Olig2 using immunoelectron microscopy and described their fine features through conventional TEM. Our results show that NG2 is expressed in the cytoplasm, endoplasmic reticulum, and intermediate filaments present in the processes of star-shaped electron-lucent cells (Figure 2A). PDGFRα, on the other hand, is expressed in small clusters in discrete regions of the plasma membrane of OPCs (Figure 2B). A2B5 is observed to be expressed in a cytoplasmic portion of OPCs which includes the endoplasmic reticulum (ER; Figure 2C). Finally, Olig2 labels the nuclei of these populations (Supplementary Figure 2).

**Figure 3:**
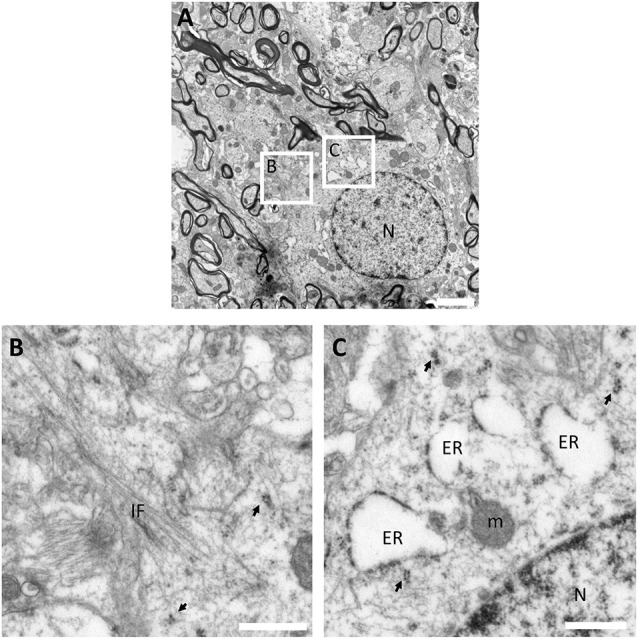
Ultrastructural characterization of OPCs in the human white matter. **(A)** Panoramic micrograph of an OPC showing a large electron-lucent star-shaped cell with electron-lucent nucleus (with abundant euchromatin) intermingled between myelinated axons in the white matter. **(B)** OPCs present abundant filamentous processes, and **(C)** an electron-lucent cytoplasm with abundant short dilated endoplasmic reticulum cisternae, mitochondria and ribosomes. The micrograph proceeds from the white matter of a 27-year-old male (PB8) fixed with 2% PFA-2.5% Glutaraldehyde. N, Nucleus; ER, endoplasmic reticulum; m, mitochondria; IF, intermediate filaments; arrows, polyribosomes. Scale bars: (**A**), 2 μm; **(B,C)**, 500 nm.

The fine structure of OPCs is very similar to that of astrocytes present in the human white matter (Supplementary Figure 1). TEM analysis shows that human OPCs can be identified as star-shaped cells with electron-lucent nucleus and non-condensed euchromatin (Figure 3A). Their soma exhibits several filamentous processes (Figure 3B) and their electron-lucent cytoplasm presents abundant short dilated endoplasmic reticulum, similar to that of classic oligodendrocytes, being also rich in mitochondria and ribosomes (Figure 3C).

### Pre-oligodendrocytes in the Human White Matter Show Scarce Organelle Variety

**Figure 4:**
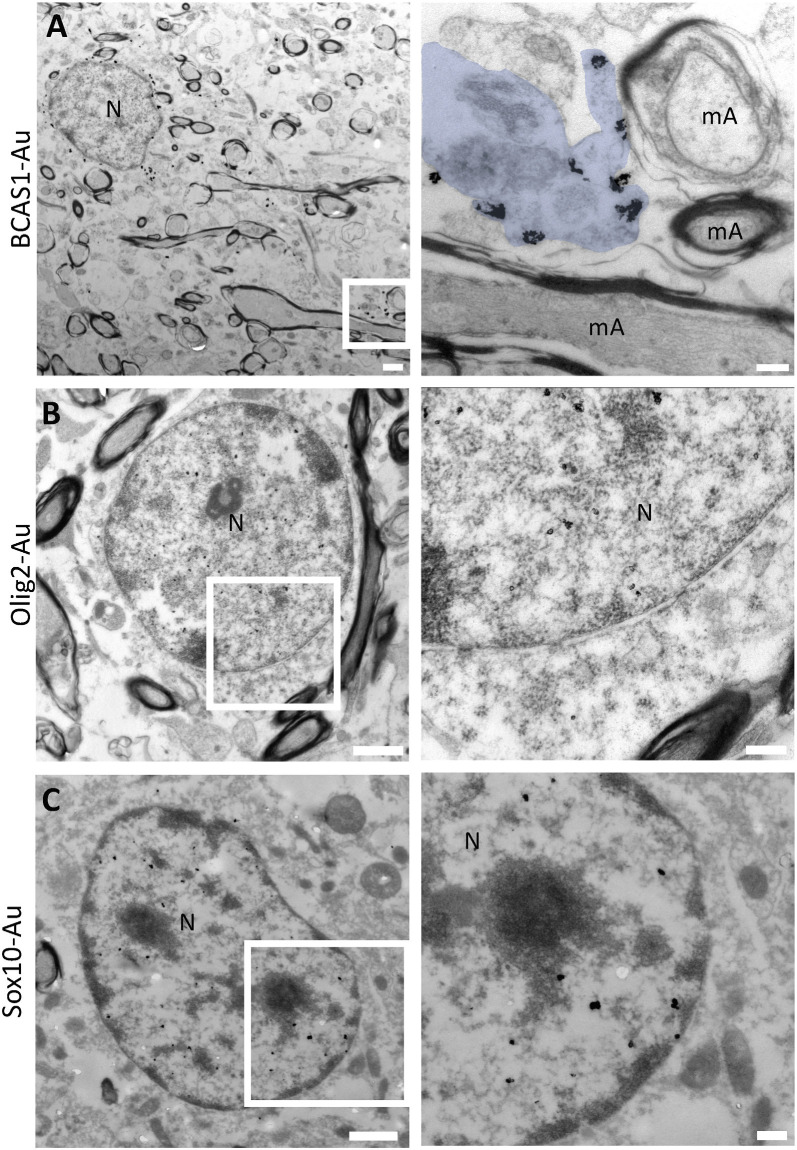
Pre-embedding immunogold labeling for pre-myelinating oligodendrocytes. **(A)** BCAS1/NABC1 labeled pre-myelinating oligodendrocyte. BCAS1 is observed in the plasma membrane of the cell and in processes in direct contact with thin myelin sheets (blue pseudocolor) in the white matter of a 2-year-old female (PB9). **(B)** Olig2 labels the uncondensed chromatin of electron-lucent cells with scant cytoplasm in the white matter of a 32-year-old male (PB16). **(C)** The transcription factor SOX10 labels the nucleus of pre-myelinating oligodendrocytes in the white matter of a 13-year-old female (PB11). N, Nucleus; mA, myelinated axon. Scale bars: panoramic micrographs: 1 μm; insets: 250 nm.

Since pre-myelinating oligodendrocytes presented a distinctive morphology in our immunofluorescence studies, we performed immunogold and conventional TEM analyses to study whether these morphological changes can be correlated with characteristic ultrastructural features. Our TEM data corroborate that the soma of pre-OLs is small, with the vast majority of its volume occupied by the nucleus. We found that the expression of BCAS1/NABC1, a novel marker of intermediate oligodendroglial maturational stages (Fard et al., 2017), is tightly associated with the plasma membrane of this population and allows the identification of cellular processes which contact thin myelin sheets (Figure 4A). On the other hand, the transcription factors Olig2 and Sox 10 are expressed in the nuclei of pre-OLs (Figures 4B,C). The distribution of both markers is similar to that of loosely condensed euchromatin within the nuclear space.

**Figure 5:**
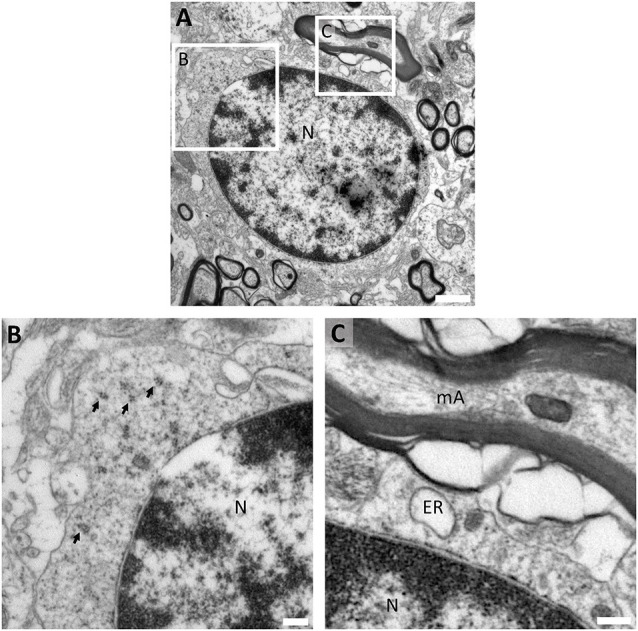
Fine structure of human pre-myelinating oligodendrocytes. **(A)** Pre-myelinating oligodendrocytes are highly ramified cells with scant electron-lucent cytoplasm and slightly condensed chromatin. **(B)** Their cytoplasm contains abundant polyribosomes, **(C)** a few small mitochondria and short dilated endoplasmic reticulum. These cells are in direct contact with myelin sheets The micrograph proceeds from the white matter of a 9-year-old male (PB2) fixed with 2% PFA–2.5% Glutaraldehyde. N, Nucleus; ER, endoplasmic reticulum; arrows, polyribosomes. T Scale bars: **(A)**, 1 μm; **(B,C)**, 250 nm.

Prior immunogold characterization of pre-OLs allowed us to further study this cell population in better-preserved conventional TEM samples. As a result, we observed that pre-OLs are highly ramified cells with scant electron-lucent cytoplasm and slightly condensed chromatin (Figure 5A). Since the cytoplasmic area is rather small, this population does not present a great organelle abundance. Polyribosomes are the most frequent organelle in pre-OLs (Figure 5B), while they also present few mitochondria and short dilated ER. Interestingly, this subset of cells displays a stretch contact with myelin sheets (Figure 5C).

### Mature Oligodendrocytes Are Electron-Dense Cells Distributed Through the White Matter

**Figure 6:**
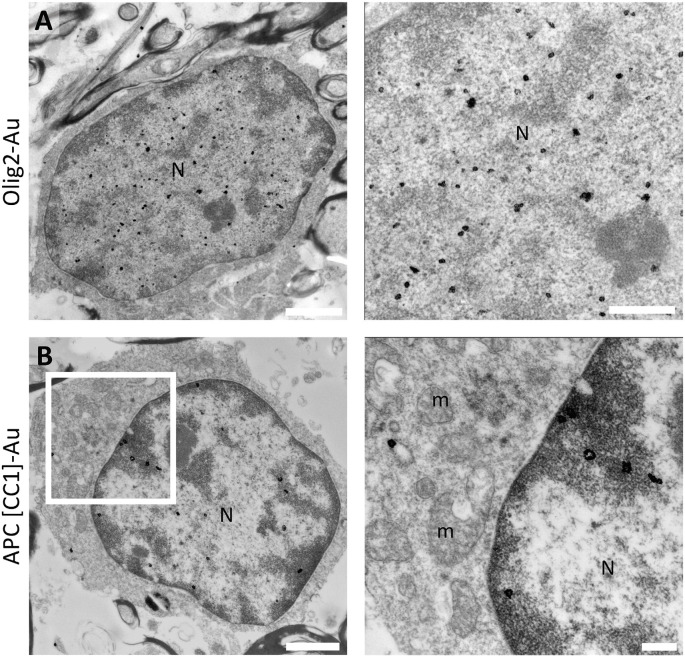
Fully differentiated oligodendrocytes display Olig2 and APC-(CC1) labeling. **(A)** Olig2 expression in mature oligodendrocytes is restricted to the uncondensed chromatin within the nucleus in the white matter of a 44-year-old male (PB12). **(B)** The RNA-binding protein quaking7 labeled with APC-(CC1) was found in the condensed chromatin portion as well as in non-condensed chromatin; faint labeling was also observed in the plasma membrane in the white matter of a 13-year-old female (PB11). N, Nucleus; m, mitochondria. Scale bars: panoramic micrographs, 1 μm; insets: 250 nm.

To gain insight into the fine structure of fully differentiated oligodendrocytes in the human white matter, we performed immunogold labeling using Olig2 and APC-(CC1) antibodies combined with standard TEM analysis. As observed in the previous stages of differentiation, Olig2 is also expressed in m-OLs. The distribution of this transcription factor is associated with non-condensed chromatin within the nucleus (Figure 6A). Conversely, the RNA-binding protein quaking7 labeled with APC-(CC1) was found associated both to euchromatin and heterochromatin as well as in the cytoplasm (Figure 6B).

**Figure 7:**
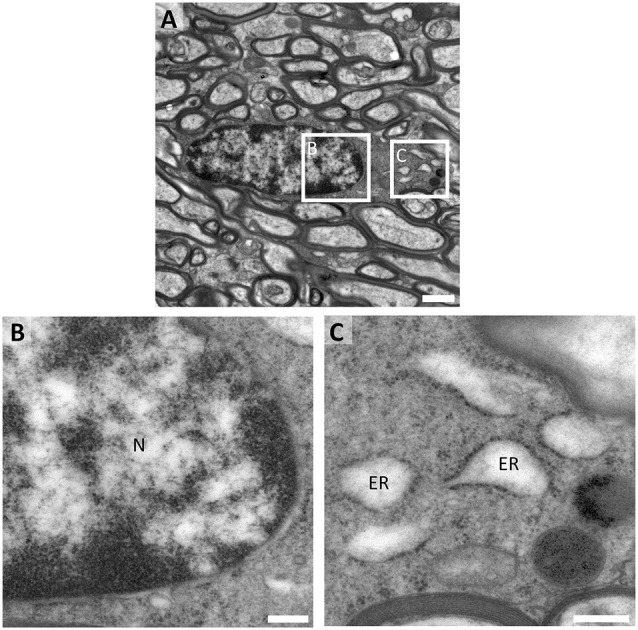
Mature oligodendrocytes are electron-dense cells in the human white matter. **(A)** Mature oligodendrocytes are small electron-dense cells with a broad distribution in the white matter. These cells are intermingled between myelin-wrapped axons. **(B)** Chromatin condensation occurs bound to the inner nuclear membrane in mature oligodendrocytes. **(C)** Cytoplasmic organization in this population displays characteristic short dilated endoplasmic reticulum, few mitochondria, dense degradation vesicles, and polyribosomes. The micrograph proceeds from the white matter of a 6-year-old male (PB3) fixed with 2% PFA-2.5% Glutaraldehyde. N, Nucleus; ER, endoplasmic reticulum. Scale bars: **(A)**, 1 μm; **(B–C)**, 250 nm.

Conventional ultrastructural characterization allowed us to describe that m-OLs are small electron-dense cells with a broad distribution in the human white matter. These cells are intermingled between myelin-wrapped axons (Figure 7A). Their chromatin is more condensed than that observed in earlier stages of oligodendroglial differentiation. Frequently, highly condensed chromatin is bound to the inner nuclear membrane (Figure 7B). m-OLs display characteristic short dilated ER, mitochondria, dense degradation vesicles, and polyribosomes (Figure 7C).

### Human Myelin-Associated Proteins Are Observed in Different Portions of the Wrapping Sheets in Pre-embedding Immunogold

**Figure 8:**
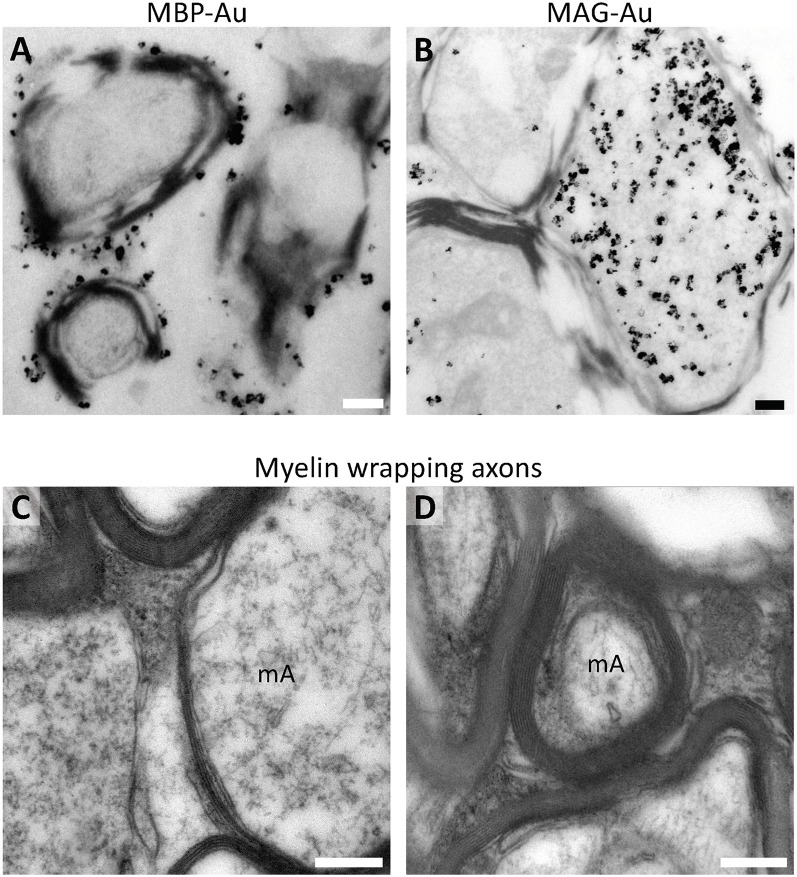
Ultrastructural analysis of myelin in the human *pre-mortem* white matter. **(A)** Pre-embedding immunogold labeling shows that MBP is located in the outermost layer of the myelinated axon in the cerebral white matter of a 5-year-old female (PB19). **(B)** MAG is observed in the internal layer of the myelinated axon in direct contact with the axon in the white matter of a 5-year-old female (PB19). **(C)** Glutaraldehyde fixation allows a better analysis of the fine structure of myelin sheets in different stages of the myelination process, allowing to discern different myelin thickness. **(D)** Furthermore, it allows the visualization of the number of myelin layers. Ultrastructural analysis reveals that myelin is tightly packed, and exhibits variable periaxonal space. **(C,D)** Micrographs proceed from the white matter of a 2-year-old female (PB9) fixed with 2% PFA-2.5% Glutaraldehyde. mA, myelinated axon. Scale bars: 250 nm.

To further investigate into the structure of human myelin structure, we sought to study the expression and localization of two well-studied myelin-associated proteins (MBP and MAG) by pre-embedding immunogold detection. Our analysis shows that MBP localizes to the outermost layer of the myelinated axon in the cerebral white matter (Figure 8A), while MAG is observed in the most internal layer of the myelinated axon in direct contact with the axon (Figure 8B). However, to study the fine structure of myelin sheets in different stages of the myelination process, conventional TEM is necessary. Our analysis unveiled that for the study of different myelin features, such as thickness (Figure 8C) or the number of myelin sheets wrapping a particular axon (Figure 8D) high-quality fixation with glutaraldehyde is required. The drawback of this fixation protocol is that it is, in many cases, non-compatible with immunogold labeling. Nevertheless, the combination of immunogold detection and conventional TEM allowed us to observe that while tightly packed, human white matter myelin presents variable periaxonal space as well as myelin thickness, which could be related to the particular type or function for each axon.

## Discussion

Oligodendrocytes and their progenitors are cells of great interest because of the several diseases related to their malfunction and the potential that this population represents in the regeneration of the central nervous system. On the other hand, ultrastructural studies in human brain tissue are scarce since most of the studies are based on post-mortem samples, in which the quality of the tissue is not optimal neither for TEM analysis nor for immunogold labeling and processing. TEM studies are highly valuable in oligodendrocyte analysis given that the gold standard to determine the remyelination rate in white matter regeneration is measured by the *g-ratio*.

The identification of different stages of morphological transformation in the oligodendroglial lineage from NG2-positive cells has been described in mouse control tissue (Sánchez-González et al., 2020). Furthermore, the generation of oligodendrocytes from OPCs has also been studied in mouse models of focal demyelination in the white matter (Menn et al., 2006). However, because of the above-mentioned technical limitations in the obtention of well-preserved human tissue, these studies are scarce.

Here we have studied the human *pre-mortem* white matter, which displays a great status of preservation, to shed light on the variation of the ultrastructural morphology of the oligodendroglial lineage combined with pre-embedding immunogold labeling for nine well-known oligodendroglial markers. Our results suggest that, in the human white matter, there are three markedly different stages of differentiation in oligodendrocyte progenitors according to their ultrastructural features and molecular markers.

The first stage of differentiation can be recognized by the expression of PDGFRα, NG2, and A2B5 (Berg and Schachner, 1982; Schnitzer and Schachner, 1982; Richardson et al., 1988; Chang et al., 2000; Rivers et al., 2008). This subset of progenitors presents ultrastructural and morphological characteristics that are very similar to astrocytes such as an electron-lucent filamentous cytoplasm, an electron-lucent nuclear content with scarcely condensed chromatin, dilated ER, mitochondria, and ribosomes. Therefore, when analyzing OPCs through TEM we recommend performing immunogold to avoid misclassification between this population and astrocytes.

The second stage of differentiation (pre-OLs) has been described as a transitory stage of maturation of oligodendrocytes. This phase is characterized by the expression of the molecular marker BCAS1/NABC1, Sox10, and Olig2 (Fard et al., 2017; Ishimoto et al., 2017). Interestingly, this population is more prominently represented in pediatric patients than in adult patients (data not shown; Fard et al., 2017). By immunofluorescence, these cells appear to be highly ramified and present scarce cytoplasmic content. TEM analysis reveals that pre-OLs present scant cytoplasmic content in which the most abundant organelle are polyribosomes and short dilated ER. The chromatin in this population is more condensed than in OPCs. The most interesting feature of pre-OLs is their contact with myelin sheaths, which conforms with the previous studies that indicate that these cells are actively synthesizing myelin (Fard et al., 2017).

The last stage of differentiation in the oligodendroglial lineage are m-OLs, which have been typically labeled with Olig2 and APC-(CC1) (Bin et al., 2016). These cells show a remarkable electron density in their cytoplasm and nucleus. The cytoplasmic portion contains short dilated endoplasmic reticulum, mitochondria, dense degradation vesicles, and polyribosomes, while the nucleus contains abundant heterochromatin associated with the inner nuclear membrane. This indicates that, as oligodendrocytes mature, their chromatin becomes more condensed and attaches to the nuclear membrane. Another remarkable feature shared by all the oligodendroglial lineage populations is that they maintain short, dilated ER through their maturation process.

Moreover, state-of–the-art studies (Bribián et al., 2020; Perlman et al., 2020) depict RNA or protein expression differences between age, sex, and status of activation or differentiation in the oligodendroglial lineage. By immunofluorescence, we observed more ramified BCAS1-positive cells. and an apparently larger number of immature oligodendrocytes in pediatric patients (data not shown). However, our results suggest that these shifts do not translate into ultrastructural differences, thus, allowing the characterization of oligodendrocytes despite the demographic differences.

Regarding the myelin that OLs synthesize, we used MBP and MAG for labeling. The first is found in the outermost portion of the myelinated axon, while the latter is found in direct contact with the axon. However, the conservation of myelin and thereby the labeling are not as precise. We suggest that myelin studies in the human brain require exquisite fixation, which implies the addition of glutaraldehyde in high concentrations (2.5%), which is a barrier to overcome for immunolabeling.

Hereby, studying the differences between the stages of differentiation in human oligodendrocytes has been of great interest because of the neuropathological implications especially in frequent disabling neurodegenerative conditions such as MS (Marques et al., 2016; Fard et al., 2017; Jäkel et al., 2019). In this pathology, the presence of immature oligodendrocytes in lesioned areas can indicate remyelination (Lucchinetti et al., 2000). Nonetheless, the cellular composition combined with the myelination/remyelination status has never been studied at an ultrastructural level, which could enhance the current knowledge and could give clues of intracellular processes within the cells in a lesion. Therefore, the fine characterization of the oligodendroglial lineage in the human brain can lead to future studies that include tissue fixed for TEM studies (i.e., glutaralehyde), that allow the combination of both myelination analysis and subcellular organelle composition for each cell type.

## Data Availability Statement

The original contributions presented in the study are included in the article/Supplementary Material, further inquiries can be directed to the corresponding author/s.

## Ethics Statement

The studies involving human participants were reviewed and approved by Research Ethics committee from the Hospital Universitari i Politècnic La Fe. Written informed consent to participate in this study was provided by the participants’ legal guardian/next of kin.

## Author Contributions

MU-N, VH-P, and JG-V conceived the original idea and supervised the experimental procedures. PP-B and AG-M performed surgical resections. PP-B selected the non-affected areas of the white matter for analysis. MU-N, RM-G, AS-T, and PG-T performed immunofluorescence, immunogold, and TEM processing. MU-N, VH-P, and JG-V wrote the manuscript. All authors contributed to the article and approved the submitted version.

## Conflict of Interest

The authors declare that the research was conducted in the absence of any commercial or financial relationships that could be construed as a potential conflict of interest.

## Supplementary Materials

SUPPLEMENTARY FIGURE 1 The ultrastructural characteristics of GFAP-positive astrocytes are similar to OPCS. **(A)** A GFAP-labeled astrocyte appears as a large electron-lucent cell (black asterisk) compared to an unlabeled oligodendrocyte (white asterisk) in the white matter. The inset shows that the GFAP silver enhanced label marks intermediate filaments in this cell. **(B)** GFAP-labeled expansions (arrows) surrounding an unlabeled OPC (white asterisk) showing the similarity of the morphology between these two populations. **(A,B)** Micrographs were obtained from the white matter of a 6-year-old male (PB3). Scale bars: panoramic micrographs, 1 μm; insets: 250 nm.

SUPPLEMENTARY FIGURE 2 OPCs express Olig2 in the nucleus. An electron-lucent cell displaying Olig2 label in the nuclear compartment in the white matter of a 27-year-old male (PB8). Scale bar: 1 μm.
