# Supplementary figures and images for: Ultrastructural Characterization of Human Oligodendrocytes and Their Progenitor Cells by Pre-embedding Immunogold
Source: Front Neuroanat. 2021 Jun 23;15:696376. doi: 10.3389/fnana.2021.696376 (PMC8262677; doi:10.3389/fnana.2021.696376)

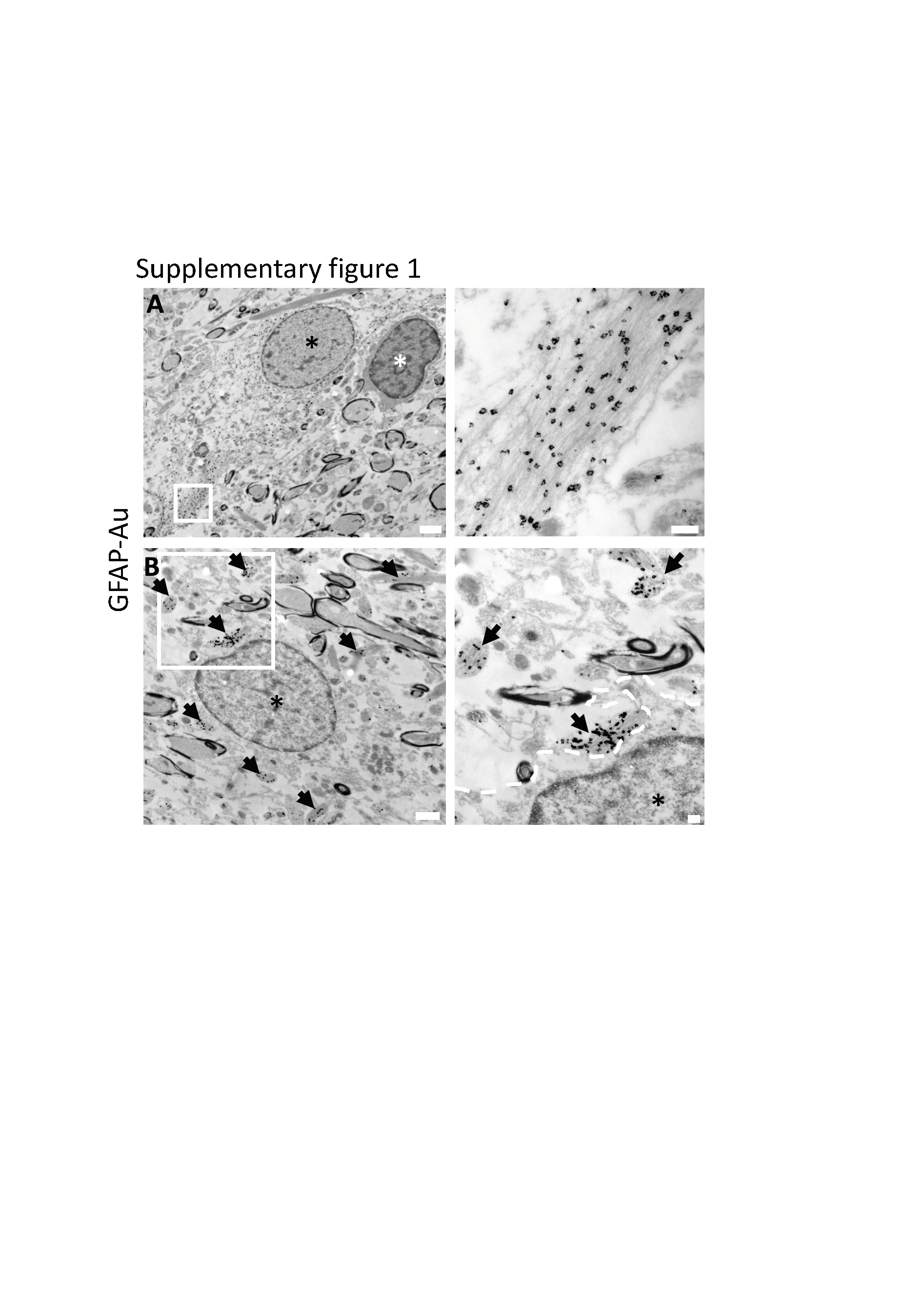

Supplement: SUPPLEMENTARY FIGURE 1 — The ultrastructural characteristics of GFAP-positive astrocytes are similar to OPCS. (A) A GFAP-labeled astrocyte appears as a large electron-lucent cell (black asterisk) compared to an unlabeled oligodendrocyte (white asterisk) in the white matter. The inset shows that the GFAP silver enhanced label marks intermediate filaments in this cell. (B) GFAP-labeled expansions (arrows) surrounding an unlabeled OPC (white asterisk) showing the similarity of the morphology between these two populations. (A,B) Micrographs were obtained from the white matter of a 6-year-old male (PB3). Scale bars: panoramic micrographs, 1 μm; insets: 250 nm. [file Image_1.TIFF]

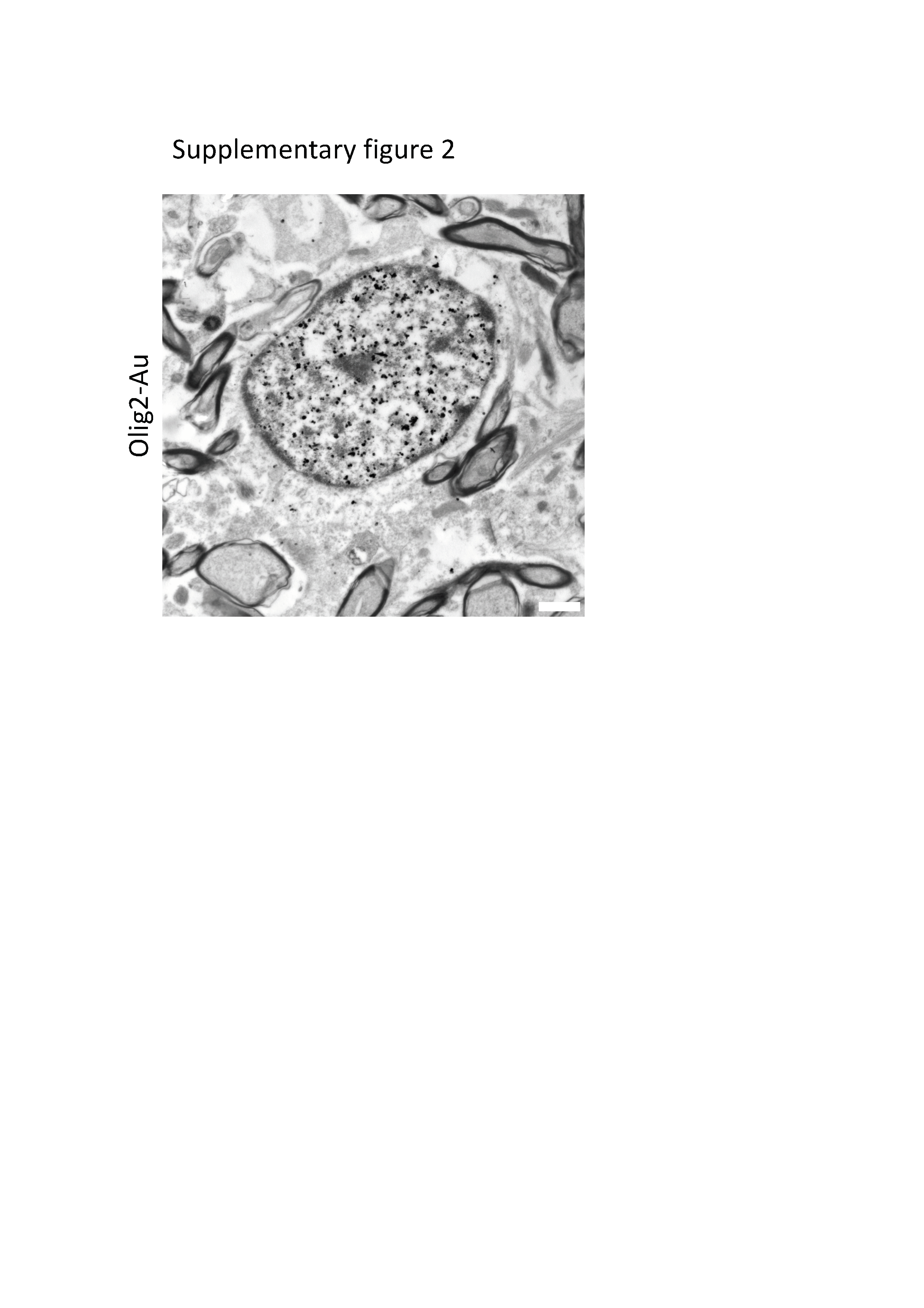

Supplement: SUPPLEMENTARY FIGURE 2 — OPCs express Olig2 in the nucleus. An electron-lucent cell displaying Olig2 label in the nuclear compartment in the white matter of a 27-year-old male (PB8). Scale bar: 1 μm. [file Image_2.TIFF]
